# Potential of extracellular vesicles in the pathogenesis, diagnosis and therapy for parasitic diseases

**DOI:** 10.1002/jev2.12496

**Published:** 2024-08-08

**Authors:** Ana Acacia Sá Pinheiro, Ana Claudia Torrecilhas, Bruno Solano de Freitas Souza, Fernanda Ferreira Cruz, Herbert Leonel de Matos Guedes, Tadeu Diniz Ramos, Miqueias Lopes‐Pacheco, Celso Caruso‐Neves, Patricia R. M. Rocco

**Affiliations:** ^1^ Instituto de Biofísica Carlos Chagas Filho Universidade Federal do Rio de Janeiro (UFRJ) Rio de Janeiro Brazil; ^2^ Rio de Janeiro Innovation Network in Nanosystems for Health‐NanoSAÚDE/Fundação Carlos Chagas Filho de Amparo à Pesquisa do Estado do Rio de Janeiro (FAPERJ) Rio de Janeiro Brazil; ^3^ Departamento de Ciências Farmacêuticas Diadema Campus, Instituto de Ciências Ambientais, Químicas e Farmacêuticas Universidade Federal de São Paulo (UNIFESP) Diadema São Paulo Brazil; ^4^ Center for Biotechnology and Cell Therapy São Rafael Hospital Salvador Brazil; ^5^ D'Or Institute for Research and Education (IDOR) Salvador Brazil; ^6^ Instituto de Microbiologia Paulo de Goés (IMPG) Universidade Federal do Rio de Janeiro (UFRJ) Rio de Janeiro Brazil; ^7^ Fundação Oswaldo Cruz (FIOCRUZ) Instituto Oswaldo Cruz (IOC) Rio de Janeiro Brazil; ^8^ Deparment of Pediatrics Center for Cystic Fibrosis and Airway Disease Research Emory University School of Medicine Atlanta Georgia USA; ^9^ National Institute of Science and Technology for Regenerative Medicine INCT‐REGENERA Rio de Janeiro Brazil

**Keywords:** Chagas Disease, extracellular vesicles, helminthiasis, leishmaniasis, malaria, toxoplasmosis

## Abstract

Parasitic diseases have a significant impact on human and animal health, representing a major hazard to the public and causing economic and health damage worldwide. Extracellular vesicles (EVs) have long been recognized as diagnostic and therapeutic tools but are now also known to be implicated in the natural history of parasitic diseases and host immune response modulation. Studies have shown that EVs play a role in parasitic disease development by interacting with parasites and communicating with other types of cells. This review highlights the most recent research on EVs and their role in several aspects of parasite‐host interactions in five key parasitic diseases: Chagas disease, malaria, toxoplasmosis, leishmaniasis and helminthiases. We also discuss the potential use of EVs as diagnostic tools or treatment options for these infectious diseases.

## 1 INTRODUCTION

Protozoan and helminthic infections represent critical public health issues due to their significant burden of morbidity and mortality worldwide, especially in tropical regions. Malaria alone accounted for over 249 million infections and 608,000 deaths in 2022 (WHO, 2023). Approximately 200,000 deaths each year are estimated to be due to neglected tropical diseases, which consist of a diverse group of 20 conditions—many parasitic—including Chagas disease, leishmaniasis and helminthiases (WHO, 2023).

Human infection and the recurring nature of these diseases can be attributed to the extraordinary ability of parasites to survive and persist within their hosts. These pathogens have evolved specialized mechanisms to gain access to host cells and evade the immune response (Torrecilhas et al., 2020). In the quest to unravel the pathogenic mechanisms of parasitic diseases, the role of EVs has become increasingly evident as a key factor (Cortes‐Serra et al., 2022; Torrecilhas et al., 2020). EVs are cell‐derived particles that contain bioactive cargoes responsible for mediating intercellular communication (Colombo et al., 2014; Fernandez‐Becerra et al., 2023; Théry et al., 2018). Across a diverse array of pathogens, including bacteria, fungi and parasites, the secretion of EVs represents a conserved mechanism that mediates inter‐pathogenic communication and modulates host‐pathogen interactions, impacting both cellular and immune host responses (Campos et al., 2015; Rizzo et al., 2020; Soares et al., 2017; Torrecilhas et al., 2012, 2020; Wang et al., 2019). By modifying cell‐signalling activities, these particles can affect the host's innate and acquired immune responses alike (Campos et al., 2015; Torrecilhas et al., 2012, 2020). Moreover, EVs orchestrate the transfer of diverse biomolecules—including proteins, nucleic acids, lipids and other bioactive compounds—across cells, and are thus inherently implicated in intracellular communication (Buzas, 2022; Fernandez‐Becerra et al., 2023; Théry et al., 2009, 2002, 2018; Yáñez‐Mó et al., 2015).

This review will summarize relevant findings concerning the role of EVs in the pathogenesis of malaria, Chagas disease, toxoplasmosis, leishmaniasis and helminthiases. The potential utility of EVs as a diagnostic and, in some cases, therapeutic tool for parasitic infections is also addressed.

### 1.1 EV function and biogenesis

EVs modulate immune response (Wolfers et al., 2001), genetic material delivery (Théry et al., 2009, 2002, 2018; Yáñez‐Mó et al., 2015) as well as cell‐to‐cell and parasite‐to‐cell interaction and communication (Soares et al., 2017; Théry et al., 2018). EVs are a heterogeneous group of lipid bilayer‐enclosed particles containing proteins, glycoconjugates, lipids, RNAs and microRNAs, among other cargo. Their release by cells occurs through outward budding of the plasma membrane or inward budding of the endosomal membrane, resulting in the formation of multivesicular bodies (MVB) (Théry et al., 2018). When a vesicle fuses with the plasma membrane of the parent cell, it releases its contents into the extracellular space through a process called exocytosis  (Colombo et al., 2014; Théry et al., 2009, 2002, 2018).

The members of the International Society for Extracellular Vesicles (ISEV) supported and endorsed two previous guidelines referred to as Minimal Information for Studies of Extracellular Vesicles (MISEV) (Théry et al., 2018), aiming to maximize the isolation, characterization and categorization of EV terms. The MISEV 2023 suggesting additional techniques for methodology and EV terminology (Welsh et al., 2024). The ISEV guideline recommends the terminology: EVs, particles released from cells, contain a lipid bilayer and do not replicate themselves. The Non‐vesicular Extracellular Particles (NVEPs) are an assembly of complex molecules released from cells with no lipid bilayer (non‐vesicular extracellular particle fraction). Another suggestion is Extracellular Particles (EPs) include all entities outside the cell (e.g., EVs and NVEPs). The EV Mimetic, artificial particles resembling EVs obtained through direct artificial manipulation, is a term preferred over ‘exosome‐like vesicle’ to avoid implications of specific biogenesis properties. The Artificial Cell‐Derived Vesicles (ACDVs), particles mimetics generated in laboratory settings because of cell disruption, such as extrusion. The Synthetic Vesicles (SVs), EV mimetics are produced de novo by molecular components or hybrid entities (fusions between liposomes and native EVs) in Small EVs (diameter < 200 nm) and Large EVs (diameter > 200 nm) should be interpreted with caution due to methodological differences.

All parasites and pathogens shedding EVs whose main function is parasite‐host interaction and modulate immune system (Campos et al., 2015; Rizzo et al., 2020; Soares et al., 2017; Torrecilhas et al., 2012, 2020). By modifying cell‐signalling activities, these particles can affect the host's innate and acquired immune responses alike (Campos et al., 2015; Torrecilhas et al., 2012, 2020). Furthermore, because they oversee the transfer of different types of components, such as proteins, nucleic acids, lipids and other physiologically active substances, across cells, these particles are inherently implicated in intracellular communication (Buzas, 2022; Fernandez‐Becerra et al., 2023).

## 2 SELECTED PARASITIC DISEASES AND THE ROLE OF EXTRACELLULAR VESICLES

### 2.1 Chagas disease

*Trypanosoma cruzi*, the protozoan parasite that causes Chagas disease, has infected 6–8 million people, with a further 5 million having uncertain infection status, which represents a major health issue worldwide (www.who.int.tdr.en/). *T. cruzi* infection occurs via skin lesions or mucosa, transmitted by insect‐vector faeces, or orally, through intake of contaminated food (e.g., açai fruit or sugarcane juice) (Lopez‐Garcia & Gilabert, 2023). The transmission can also occur via blood transfusions, organ transplants and vertical transfer from mother to child during pregnancy or delivery.

#### 2.1.1 EVs and Chagas disease pathogenesis

*T. cruzi* expresses several plasma membrane molecules that are involved in host cell invasion by modulating the immune system and evading host defences. These include mucin‐like glycoproteins, mucin‐associated surface proteins (MASP), and trans‐sialidase (gp85/TS) (Abuin et al., 1996a, 1989, 1996b; Almeida & Gazzinelli, 2001; Buscaglia et al., 2004; Campos et al., 2001; Procópio et al., 2002), which are crucial for inducing cytokine and NO production (Almeida et al., 2000, 1999; Camargo et al., 1997; Monteiro et al., 2006; Schenkman et al., 1993). Mucin‐like glycoproteins, particularly abundant on trypomastigote surfaces, trigger proinflammatory responses, attributed to specific unsaturated fatty acids in the glycosylphosphatidylinositol (GPI) anchor (Almeida et al., 2000, 1999; Camargo et al., 1997; Fernandez‐Becerra et al., 2023).

**FIGURE 1:**
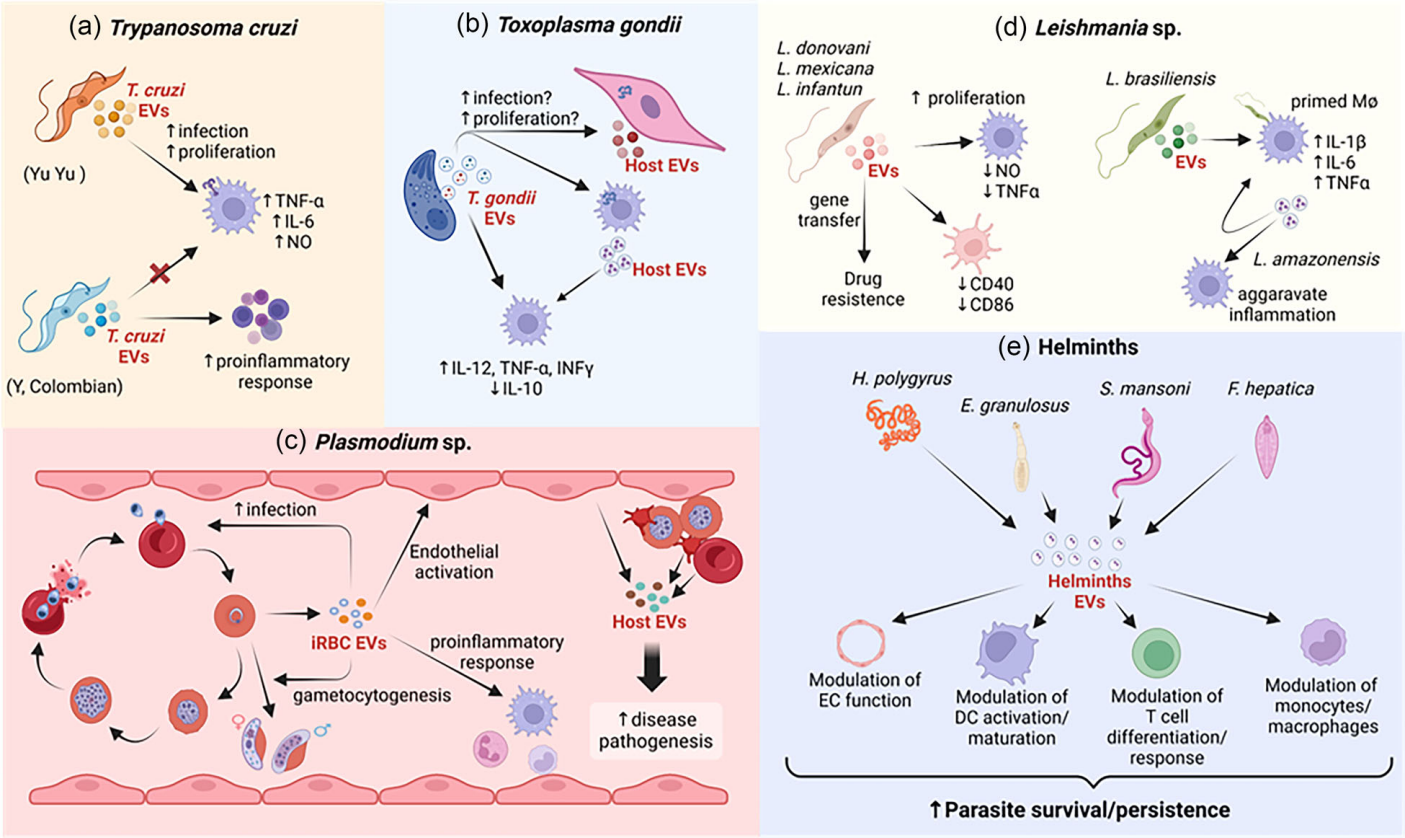
An overview of the contribution of EVs to the pathogenesis of parasitic diseases. (a) *Trypanosoma cruzi*: parasite‐derived EVs modulate the host immune system to promote invasion via TLR‐2. In macrophages, EVs derived from the most virulent strain (YuYu) increase parasite intracellular proliferation and the host proinflammatory response, followed by the release of greater amounts of TNFα, IL‐6 and NO, which is not observed with less virulent strains (Y and Colombian). Instead, EVs isolated from Y and Colombian strains induce a proinflammatory response in splenocytes during the chronic phase of *T. cruzi* infection. (b) *Toxoplasma gondii*: Host cells infected with *T. gondii* produce EVs that change both host and neighbouring cells’ proliferation mechanisms. *T. gondii* tachyzoite‐derived EVs contain proteins used for invasion and proliferation in the host cell, suggesting a role in facilitating infection. Tachyzoite‐derived EVs induce macrophage activation and a proinflammatory response, increasing levels of IL‐12, TNFα and INFγ while reducing IL‐10. Moreover, EVs from *T. gondii*‐infected macrophages carry pathogen‐associated molecular patterns (PAMPs), thus driving activation and a proinflammatory phenotype in other macrophages in the vicinity. (c) *Plasmodium* sp.: During infection, plasma levels of EVs are elevated and correlate directly with severe disease. EVs derived from infected erythrocytes (iRBC‐EVs) influence parasite invasion and survival. These EVs also induce gametocyte differentiation, supporting parasite survival and lifecycle progression. It is also recognized that iRBC‐EVs promote endothelial cell activation, increasing parasite sequestration and induce a proinflammatory response by activating monocytes, lymphocytes and neutrophils. EVs can also be secreted by host cells, including endothelial cells, erythrocytes and platelets, thus contributing to disease aggravation. (d) *Leishmania* sp.: Parasite‐derived EVs subvert the host immune response, promoting parasite survival by a mechanism that induces reduction of the microbicidal agents NO and TNFα. Moreover, *L. donovani*‐derived EVs downmodulate the expression of CD86 and CD40 in antigen‐presenting cells (DCs). Parasite EVs promote gene transfer and are associated with drug resistance. In *L. brasiliensis* infection, parasite‐derived EVs increase the proinflammatory response by priming macrophages to increase the production of IL‐1β, IL‐6 and TNF‐α upon infection. Macrophages infected with *L. amazonensis* generate EVs that can intensify the immune response, aggravating disease. (e) Helminths: EVs derived from different helminth species directly induce endothelial cell activation and modulate the host immune response to promote survival and persistence of infection. Created with BioRender.com.

Epimastigote (da Silveira et al., 1979) and trypomastigote (Gonçalves et al., 1991) forms of *T. cruzi* release EVs, with those released by trypomastigote forms containing molecules from the parasite's plasma membrane. Various groups have since demonstrated *T. cruzi*‐released EVs at different stages of the parasite's life cycle (Bayer‐Santos et al., 2013; Cestari et al., 2012; Ramirez et al., 2018, 2017; Torrecilhas et al., 2012, 2009, 2020). *T. cruzi*‐released EVs can modulate the host immune system and enhance the parasite's invasion via host cell expression of toll‐like receptor 2 (TLR2) (Cronemberger‐Andrade et al., 2020). Our group has shown that Y and YuYu strains of trypomastigotes release EVs with differences in glycoconjugate expression, and YuYu strain derived EVs increase the intracellular proliferation of parasites in macrophages (Ribeiro et al., 2018). In contrast, Y and Colombian strain‐released EVs do not induce the production of tumour necrosis factor (TNF)‐α, NO, or interleukin (IL)−6 in murine macrophages when compared to YuYu and CL‐14 strains. During the chronic phase of *T. cruzi* infection, Y and Colombian strain isolated EVs increase the pro‐inflammatory response in splenocytes from C57BL/6 mice (Ribeiro et al., 2018). Blood trypomastigotes from different parasites spontaneously release EVs of approximately 20–200 nm diameter, and different amounts and types of EVs correlate with different *T. cruzi* infectivity. An overview of the contribution of EVs to the pathogenesis of *T. cruzi* is shown in Figure 1.

EVs collected by gel filtration mostly include mucin‐like glycoproteins containing modified α‐galactosyl residues, which are highly immunogenic. The α‐galactosyl‐enriched EVs induce proinflammatory responses in murine macrophages via a TLR‐2‐dependent structure. In addition, proteomic analysis of these EVs showed multiple members of the gp85/TS superfamily. These proteins are likely to have a role in parasite adherence to host cells, thus influencing infectivity, especially with more virulent strains. Indeed, some of these proteins, such as (TS) enzymes, allow host cells to connect, invade and escape host defences, protecting the parasite from host complement and antibodies while regulating host immune‐mediated and apoptotic responses. Although TS is differentially expressed in strains with different characteristics, it is downmodulated in avirulent strains. Other members of the TS/gp85 superfamily found in EVs, though lacking enzymatic activity, may also have significant effects on host‐cell interactions. EVs isolated from the YuYu strain (more virulent) showed lower infection rates but a higher number of intracellular parasite multiplication in macrophages compared to those from the Y strain (Ribeiro et al., 2018).

#### 2.1.2 EVs and diagnosis of chronic Chagas disease

Several studies have demonstrated that EVs play a crucial role in the pathophysiology of chronic Chagas disease as biomarkers, modulating the immune system and affecting progression of the infection, especially in immunosuppressed patients (Fernandez‐Becerra et al., 2023; Madeira et al., 2021, 2022, 2024). Measurement of EV concentrations in bodily fluids of Chagas patients have yielded controversial results (Cortes‐Serra et al., 2022, 2020; Fernandez‐Becerra et al., 2023), with some studies reporting that treated Chagas patients exhibit reduced levels of EVs (Fernandez‐Becerra et al., 2023; Madeira et al., 2021) and different cytokine profiles (Cortes‐Serra et al., 2022; Fernandez‐Becerra et al., 2023). This reduction in EV secretion could potentially be associated with impaired immune responses to the parasite, compromising host defence mechanisms. EVs have also been associated with chronic inflammation and oxidative stress, particularly in patients with cardiac involvement, and have been implicated in disease severity and progression (Fernandez‐Becerra et al., 2023). EVs isolated from patients with indeterminate‐form Chagas disease or ECG changes have been found to increase interferon (IFN)‐γ production, thus contributing to sustained host inflammation (Madeira et al., 2021). EVs isolated from Chagas patients were also shown to contain α‐galactosyl (mucin) and TS (Fernandez‐Becerra et al., 2023; Madeira et al., 2022). Key findings concerning the expression of glycoconjugates and immunomodulatory effects of *T. cruzi*‐derived EVs are described in Table 1.

**TABLE 1:** Summary of studies on the expression of glycoconjugates and immunomodulatory effects of *T. cruzi*‐derived EVs.

*T. cruzi* strain(s)	Key findings	Reference
Y	The first evidence that *T. cruzi* releases EVs from epimastigotes (invertebrate stage).	da Silveira et al. (1979)
Y, CA1, RA and YuYu	EVs shed by trypomastigotes (vertebrate stage) contain proteins (70–150 kDa). Most surface antigens were released in the form of plasma membrane vesicles with sizes ranging from 20 to 80 nm.	Gonçalves et al. (1991)
Y and YuYu	EVs released by trypomastigotes (human stage) contain Tc85 (glycoconjugate).	Abuin et al. (1996a, 1996b), Abuin et al. (1989)
Y (Abuin et al., 1996a, 1996b; Abuin et al., 1989)	Pre‐inoculation of mice with EVs before *T. cruzi* infection led to severe heart pathology, intense inflammation and increased amastigote nests. Analysis after 15 days showed a predominance of CD4^+^ T‐cells and macrophages, reduced CD8^+^ T‐cells, and decreased anti‐iNOS‐labelled areas. Higher IL‐4 and IL‐10 mRNA levels in hearts, increased IL‐10, and reduced NO in splenocytes are observed. Neutralizing anti‐IL‐10 or anti‐IL‐4 antibodies prevented EV‐induced effects, suggesting that *T. cruzi*‐derived EVs contributed to tissue parasitism and inflammation by stimulating IL‐4 and IL‐10 production.	Torrecilhas et al. (2009)
Silvio X10/6	EVs from epimastigotes, metacyclic trypomastigotes and tissue culture trypomastigotes form complexes on the parasite surface with complement C3 convertase, stabilizing and inhibiting it, leading to increased parasite survival. TGF‐β‐bearing EVs from monocytes and lymphocytes facilitate rapid *T. cruzi* cell invasion, aiding in evasion of complement attack. In vivo, *T. cruzi* infection correlated with elevated EV levels in mouse plasma, and exogenous EVs increased *T. cruzi* parasitemia and evasion of host innate immunity.	Cestari et al. (2012)
Y, YuYu, Colombiana and CL‐14	EVs induced cytokine production, particularly IL‐10, in chronically infected mice. High IL‐10 frequency was observed in CD4^+^ and CD8^+^ T‐cells, while B‐cells showed a mixed cytokine profile with TNF‐α and IL‐10. Dendritic cells produced TNF‐α upon EV stimulation. Surface polymorphisms in the vesicles may play a crucial role in immunopathologic events during both early infection and the chronic phase.	
Dm28c	EVs released from epimastigotes contain small RNAs originating from rRNA, tRNA, sno/snRNAs and protein‐coding sequences.	Fernandez‐Calero et al. (2015)
PAN4	EVs from trypomastigotes in *T. cruzi*‐infected mice shown by epitope and suppression of complement lysis assays. The particles caused an associated immune response. In addition, *T. cruzi*‐infected BALB/c mice showed a humoral immune response (IgM).	De Pablos et al. (2016)
PAN4	MASP proteins in EVs function as carriers for immature and damaged proteins, causing the development of circulating immune complexes. These complexes could be used as indicators of digestive problems in clinical forms of Chagas disease.	Díaz Lozano et al. (2017)
Y	EV pre‐inoculation of mice before *T. cruzi* infection reduced plasma nitric oxide and cytokine production while inducing lipid body formation. EVs affected macrophages, enhancing parasite internalization and trypomastigote release while modulating prostaglandin E2 production.	Lovo‐Martins et al. (2018)
Y and YuYu	EVs from trypomastigotes carried the virulence factors trans‐sialidase (TS) and cruzipain. EVs resulted in lower infection but higher intracellular parasite load in J774 macrophages. Conversely, YuYu EVs caused higher infection of host cells than the Y strain, suggesting strain‐specific quantitative and qualitative differences in EVs and secreted proteins correlating with infectivity and virulence.	Ribeiro et al. (2018)
Bug2149 cl10	Interaction of EVs released by epimastigotes with *Rhodnius prolixus and Triatoma infestans*. After 49–50 days, no significant influence on parasite levels in vector guts was seen when insects were fed before epimastigote infection. Pre‐feeding with EVs, on the other hand, delayed parasite migration to the rectum in *R. prolixus* after 21–22 days, implying a possible role for *T. cruzi*‐derived EVs in early infection events in the invertebrate host.	
Pan4	EVs from trypomastigotes led to increased cell permeability and elevated intracellular Ca^2+^ levels, impacting actin cytoskeleton dynamics, and arresting the cell cycle at G0/G1. These changes facilitated host cell invasion and enhanced the percentage of cell parasite infection.	Retana Moreira et al. (2019, Ribeiro et al. (2018))
Y	EVs from THP‐1 cells infected with trypomastigotes were studied. EVs interacted with TLR2 and increased the percentage of infected cells. EVs from *T. cruzi*‐infected macrophages induced NF‐κB translocation, altering gene expression of proinflammatory cytokines and STAT‐1/STAT‐3 signalling pathways. EVs induce the host inflammatory response during infection.	Cronemberger‐Andrade et al. (2020)
Pan4	EVs from epimastigotes and trypomastigotes exhibited distinct adhesion capacity, with trypomastigote EVs adhering more strongly. Surface modification occurred throughout the life cycle of *T. cruzi*, altering the physicochemical composition of EVs. Such differences affected the ability of EVs to participate in cell communication within distinct infection environments.	Retana Moreira et al. (2021)
Y	EVs from trypomastigotes induced proinflammatory activity and enhanced iNOS, Arg‐1, IL‐12 and IL‐23 gene expression in activated BMDM under diverse stress conditions. The release mechanism is dependent on membrane shape and parasite integrity. Stress did not affect EV function, demonstrating that physiological adaptability to environmental changes is possible.	Vasconcelos et al. (2021)
Human study	EVs from the plasma of chronic Chagas disease patients were studied. Biomarkers to determine response to therapy early in patients with chronic Chagas disease will be needed. The authors examined plasma‐derived EVs from a heart transplant patient with chronic Chagas disease and demonstrated the approach's potential for identifying such biomarkers.	Cortes‐Serra et al. (2020)
Human study	*T. cruzi* antigens and conventional human exosomal markers (CD9, CD63, CD81 and CD82) were found in plasma‐derived EVs from chronic Chagas disease patients. EVs from these patients contained both *T. cruzi* antigens and human exosomal markers. Future biomarker identification investigations in Chagas disease by establishing a feasible method for isolating total circulating EVs.	Madeira et al. (2021), Madeira et al. (2022)
Human and parasite study	*T. cruzi‐*infected cells and trypomastigotes released EVs that were involved in cell‐to‐cell communication and altered the host immunological response.	Cortes‐Serra et al. (2020)
Pan4	Analysed the expression of Rho‐GTPases (RhoA, Rac1 and Cdc42) after EV‐cell interaction, revealing a downregulation of the genes after 4 h. Additionally, EVs were found to play a protective role against apoptosis and increase CSNK1G1 expression. EV‐cell interactions suggest a role for EVs as virulence factors.	Cornet‐Gomez et al. (2023)
Y	The NAIP and NLRC4 proteins were required for the release of IL‐1 and NO during *T. cruzi* infection, and their absence caused macrophages to become receptive to parasite multiplication. Nlrc4^−/−^ and Nlrp3^−/−^ macrophages have similar reduced responses, indicating that these inflammasomes play non‐redundant roles during infection. Inflammasomes were activated by live trypomastigotes rather than soluble antigens or EVs. Cathepsin inhibition prevented caspase‐1 cleavage and IL‐1 and NO release, mimicking the phenotype seen in Nlrc4^−/−^ and Nlrp3^−/−^ double knockout macrophages. These findings emphasize the NAIP/NLRC4 inflammasome's critical role in macrophage responses to *T. cruzi* infection, expanding its roles beyond bacterial infections.	Amaral et al. (2023)
Y	RAW 264.7 macrophages exposed to EVs and then infected with *T. cruzi* trypomastigotes generated less NO and had a higher number of trypomastigote forms internalized in the cell than controls primed with acetylsalicylic acid, a dual COX inhibitor, before exposure to EVs. Control macrophages showed an increase in NO generation and a decrease in trypomastigote uptake. EVs influenced macrophage responses in favour of *T. cruzi*, and COX played a role in EV effects.	Dos Santos et al. (2023)

#### 2.1.3 EVs in prevention and treatment of Chagas disease

One front of action for the prevention and control of Chagas disease is the production of vaccines. Vaccines for Chagas disease could prevent infection itself or prevent progression of established disease to cardiac and digestive manifestations in already infected patients. Although efforts have been made to develop safe and effective vaccines for Chagas disease, to date no candidate has provided full protective immunity (Dumonteil & Herrera, 2021). In this context, immunization studies using proteins accumulated in parasite derived EVs have been explored as a novel approach. For instance, immunization of mice with synthetic MASP (a parasite virulence factor delivered by *T. cruzi*‐produced EVs) conjugated to keyhole limpet hemocyanin increased survival and reduced parasite load in the heart, liver and spleen upon infection. This finding correlated with increased production of neutralizing antibodies and a protective cytokine response against infection, suggesting MASP proteins may be good vaccine candidates (Serna et al, 2014). The TS family of proteins, as well as the *T. cruzi* trypomastigote alanine, valine and serine (TcTASV‐C) protein, which plays a role in immune evasion and host–parasite interactions, are also found in *T. cruzi*‐derived EVs. With some formulations, immunization of mice followed by *T. cruzi* challenge resulted in antibody production, but with no significant impact on survival (Tandoh et al., 2024). Together, these findings reveal the need for continued exploration of the therapeutic potential of EVs in Chagas disease.

### 2.2 Malaria

Malaria is a life‐threatening disease caused by parasites from the genus *Plasmodium*. In the most recent World Malaria Report, WHO recorded 608,000 deaths in 2022 (WHO, 2023). Five *Plasmodium* species can cause malaria in humans, with *P. vivax* being the dominant malaria parasite in most countries, while the highest lethality is associated with *P. falciparum*, which is most prevalent on the African continent (Sierro & Grau, 2019).

In *P. falciparum* infection, 1%–2% of cases lead to severe disease, the manifestations of which include severe anaemia, pulmonary edema with respiratory distress, acute kidney injury and cerebral malaria (CM), alone or in association (Idro et al., 2010; White, 2022).

Cerebral malaria is a neurological complication associated with convulsions and unrousable coma (White, 2022), which may lead to death (15%–25% of infected patients) or long‐lasting neurological sequelae (Schiess et al., 2020). The mechanisms of CM pathogenesis are not completely understood and, in recent decades, interest in the role of EVs as mediators of long‐distance cell‐cell communication has grown substantially (Campos et al., 2010; Coltel et al., 2006; Combes et al., 2006) (Table 2).

**TABLE 2:** Summary of major studies reporting the characterization and functions of EVs produced during malaria.

*Plasmodium* spp.	Key findings	Reference
*P. falciparum* (human study)	Plasma samples of Malawian children infected with *P. falciparum* had a 6‐fold increase in the levels of EVs from endothelial cells with CM compared to non‐CM severe malaria.	Combes et al. (2004)
*P. berghei* ANKA (mouse model)	Genetic (ABCA1 knockout mice) interference of EV production protected mice from CM and prevented mortality.	Combes et al. (2005)
*P. falciparum* (culture)	Platelet‐derived EVs had a role in iRBC cytoadherence to human brain endothelium.	Faille et al. (2009)
*P. falciparum* (human study)	Increased levels of circulating EVs in CM patients infected with *P. falciparum* from Cameroon, compared to non‐cerebral severe or uncomplicated malaria. Platelet‐derived EVs correlated positively with the severity of CM.	Mfonkeu et al. (2010)
*P. vivax* (human study)	*P. vivax*‐infected patients had increased levels of plasma circulating EVs when compared to healthy age‐matched malaria‐unexposed controls. The main sources of EVs during *P. vivax* infection were platelets, erythrocytes, and leukocytes. Platelet‐derived EVs increased linearly with acute inflammatory symptoms.	Campos et al. (2010)
*P. falciparum, P. vivax, P. malariae* (human study)	High levels of circulating EVs were found in the blood of patients infected with *P. falciparum*, *P. vivax* or *P. malariae* when compared to noninfected patients. The highest level of circulating EVs was found in *P. falciparum* severe malaria patients compared to the other infected groups. Antimalarial treatment decreased circulating EV levels in patients infected with *P. vivax* and *P. malariae*, but not with *P. falciparum*.	Nantakomol et al. (2011)
*P. falciparum* (human study)	Demonstration of a strict correlation between ABCA1 gene promoter polymorphisms and augmented plasma EV levels and disease complications in human malaria patients.	Sahu et al. (2013a)
*P. falciparum* (human study)	*P. falciparum*‐infected patients had high levels of EVs derived from different cell types (endothelial cells, platelets and erythrocytes) and this correlated with disease severity and TNF production.	Sahu et al. (2013b)
*P. falciparum* (culture)	EVs released during the erythrocytic cycle contained both host and parasite proteins, in particular parasite antigens involved with invasion. EVs modulated human primary macrophages and neutrophil responses. EVs stimulated in iRBCs the gametocytogenesis in a dose‐dependent manner.	Mantel et al. (2013)
*P. falciparum* (culture)	EVs producing during the *P. falciparum* erythrocytic cycle were used for cell‐cell communication between parasite populations and induced gametocytogenesis.	Regev‐Rudzki et al. (2013)
*P. berghei* ANKA (mouse model)	Fluorescently labelled EVs from donor‐infected mice with CM were adoptively transferred into noninfected recipient mice. EVs activated endothelial cells and induced CM‐like brain and lung pathology.	El‐Assaad et al. (2014)
*P. falciparum* (culture)	iRBC‐derived EVs contained miRNA‐Argonaute2 complexes that modulated endothelial cell activity and increased vascular permeability.	Mantel et al. (2016)
*P. berghei* ANKA (mouse model)	Analysis of protein cargo of plasma‐derived EVs in an experimental CM model revealed 60 differentially abundant host proteins when compared to EVs from non‐infected mice.	Tiberti et al. (2016)
*P. falciparum* (culture)	iRBC‐derived EVs containing parasitic small RNA and genomic DNA were internalized by monocytes to engage cytosolic receptors. The signalling response induced monocyte activation and type 1 IFN production through a STING‐dependent manner, as well as changed monocyte subset from classical to intermediate.	Khowawisetsut et al. (2023), Sisquella et al. (2017)
*P. falciparum* (culture)	Comparative study using EVs from the culture medium of normal or *P. falciparum*‐infected RBCs. This study revealed a much larger number of EVs in the culture of iRBCs than of normal RBCs. In iRBCs‐derived EVs, human Argonaute2 (hAgo2) was bound to hundreds of miRNAs. The hAgo2‐miRNA complexes were transferred into the parasites to control the expression of PfEMP1, a parasite virulence factor involved with parasite sequestration and disease severity.	Wang et al. (2017)
*P. falciparum* (culture)	EVs from early ring‐stage iRBCs contain PfEMP1. Primary human monocytes stimulated with early ring‐stage iRBC‐EVs released low levels of inflammatory cytokines. Differently, iRBC‐EVs from parasites deficient in PfEMP1 induced more gene expression changes and affected pathways involved in defence response, stress response and cellular response to cytokines.	Sampaio et al. (2018)
*P. falciparum* (culture)	EVs from cultured iRBCs contained several types of human and plasmodial regulatory RNAs (about 120 plasmodial RNAs, including mRNAs coding for exported proteins and proteins associated with drug resistance, as well as noncoding RNAs, including rRNAs, small nuclear RNAs and tRNAs).	Babatunde et al. (2018)
*P. falciparum* (culture)	EVs from *P. falciparum‐*infected erythrocytes were more efficiently internalized by infected and non‐infected RBC when compared to EVs from naive erythrocytes. iRBC‐EVs loaded with the antimalarials atovaquone and tafenoquine inhibited the erythrocytic development of *P. falciparum* in vitro better than iRBC‐EVs alone.	Borgheti‐Cardoso et al. (2020)
*P. falciparum* (culture)	*P. falciparum*‐derived EVs impaired erythropoiesis to ensure gametocyte maturation.	Neveu et al. (2020)
*P. vivax* (human study)	Plasma‐derived EVs from patients infected with *P. vivax* contained parasite proteins that were taken up by human spleen fibroblasts, leading to ICAM‐1 upregulation in an NF‐κB‐dependent manner.	Toda et al. (2020)
*P. vivax* (human study)	Plasma‐derived EVs from patients from Thailand infected with *P. vivax* or *P. falciparum* contained species‐specific miRNAs and had a role in disease onset through the regulation of specific target genes, including genes associated with adherens junction and the TGF‐β pathways.	Ketprasit et al. (2020)
*P. falciparum* (culture)	EVs from *P. falciparum* cultures were captured differently by monocytes and macrophages.	
*P. falciparum* (culture)	EVs from *P. falciparum*‐infected erythrocyte cultures contained assembled and functional 20S proteasome complexes capable of modulating the mechanical properties of naive human RBCs, priming them to infection and thus favouring parasite growth.	Dekel et al. (2021)
*P. falciparum* (culture)	*P. falciparum*‐derived EVs internalized by monocytes induced inhibition of CXCL10 synthesis by a mechanism that involves RNA cargo delivery that triggers RIG‐1, leading to HUR1 binding to an AU‐rich domain of the CXCL10 3′‐UTR.	Ofir‐Birin et al. (2021)
*P. vivax* (human study)	Proteome analysis of plasma‐derived EVs isolated from human‐liver chimeric mice (FRG huHep mice) infected with *P. vivax* identified liver‐stage parasite proteins indicative of infection.	Gualdron‐Lopez et al. (2022)
*P. falciparum* (culture)	Characterization of two distinct subpopulations of EVs from *P. falciparum*‐infected RBCs: small EVs containing complement‐system proteins and large EVs enriched in proteasome subunits. Both EV subpopulations were able to fuse to the plasma membrane, but the smaller EVs showed better fusion capability at early endosomal conditions than the larger ones.	Abou Karam et al. (2022)

#### 2.2.1 EVs and malaria pathogenesis

Malaria‐causing parasites use EVs to transfer their molecules to the host cells, a mechanism that significantly contributes to parasite survival and successful infection (Opadokun & Rohrbach, 2021). EVs released from *P. falciparum*‐infected erythrocytes have been considered important elements for gene transfer within the parasite population. Babatunde et al. (2018) described small RNAs as cargoes in cultured infected red blood cells (iRBC)‐derived EVs. They found about 120 plasmodial RNAs, among them mRNAs coding for exported proteins and proteins associated with drug resistance, as well as non‐coding RNAs, including rRNAs, small nuclear RNAs and tRNAs (Babatunde et al., 2018). Neveu et al. (2020) subsequently demonstrated that these EVs hinder erythropoiesis to ensure gametocyte maturation. This could be an approach used by *Plasmodium falciparum* to escape the host environment and evade both antimalarial treatment and the immune system, allowing it to survive and advance through its life cycle. Infected red blood cells release EVs with functional 20S proteasome complexes, which alter the mechanical properties of healthy red blood cells, making them more susceptible to infection. Furthermore, the concept of a parasite ‘decision sensing system’ controlled by CXCL10 concentration has been proposed, whereby high levels of CXCL10 lead *P. falciparum*‐derived EVs to inhibit synthesis of this cytokine, promoting the parasite's growth and survival (Dekel et al., 2021; Ofir‐Birin et al., 2021; Regev‐Rudzki et al., 2013).

EVs have emerged as a significant aspect of the immunopathological cascade of malaria, playing various roles in disease progression and severity. EVs released by infected red blood cells (iRBCs) exhibit proinflammatory effects, activating immune cells such as macrophages and neutrophils. These EVs induce CD40 upregulation and TNF production in macrophages via the MyD88/TLR4 receptor axis, as well as activate monocytes, leading to type 1 interferon production through a STING‐dependent mechanism. Additionally, they alter the monocyte subset from classical to intermediate. Interestingly, different immune cells take up EVs differentially, suggesting cell‐specific mechanisms of cargo release after internalization. EVs contain miRNA‐Argonaute2 complexes that modulate endothelial cell activity and increase vascular permeability. They contribute to endothelial cell activation, facilitating the sequestration of iRBCs in the microvasculature (Couper et al., 2010; Mantel et al., 2013). In a murine model, plasma EVs produced during infection induced cerebral malaria‐like pathology when transferred to uninfected mice. Genetic or pharmacological interventions targeting EV production protect against cerebral malaria and mortality. Additionally, there is a correlation between *ABCA1* gene promoter polymorphisms and plasma EV levels in malaria patients, suggesting a potential genetic susceptibility to severe disease (Combes et al., 2005; Khowawisetsut et al., 2023; Sahu et al., 2013a; Sisquella et al., 2017).

Although *P. vivax* is considered less clinically devastating than *P. falciparum*, it can still cause severe disease (Baird, 2007; Rahimi et al., 2014). A unique characteristic of *P. vivax* infection is the formation of hypnozoites, the latent liver stages that cause relapsing infection. Hypnozoites are clinically undetectable and represent a crucial obstacle to malaria eradication (Krotoski, 1985). In 2022, a study demonstrated that EVs derived from *P. vivax*‐infected hepatocytes carry specific proteins from hypnozoites. This study proposed circulating plasma EVs as biomarkers of latent *P. vivax* liver infection in a mouse model (Gualdron‐Lopez et al., 2022). In infected humans, parasite protein‐loaded EVs are taken up by spleen fibroblasts, leading to ICAM‐1 upregulation in an NF‐κB‐dependent manner. This mechanism is associated with specific adhesion properties of *P. vivax*‐infected reticulocytes which are still a matter of investigation (Fernandez‐Becerra et al., 2020; Toda et al., 2020). The main findings concerning EV roles in malaria pathogenesis are summarized in Figure 1.

#### 2.2.2 EVs and malaria diagnosis

In malaria, EVs can be derived from various cell types, including platelets, iRBCs, endothelial cells and immune cells, such as monocytes and lymphocytes (Combes et al., 2004; Faille et al., 2009; Mantel et al., 2016; Mfonkeu et al., 2010; Nantakomol et al., 2011). Regardless of the *Plasmodium* species, during infection, the number of EVs is increased in plasma and other bodily fluids, as shown in different models (Coltel et al., 2006; Mantel & Marti, 2014). A study performed in Malawian children revealed a 6‐fold increase in the levels of endothelial cell‐derived EVs in plasma samples of CM patients compared with those with non‐CM severe malaria (Combes et al., 2004). These findings suggest a strong correlation between high levels of EVs and the occurrence of CM. RBC‐derived EVs were increased in patients with falciparum malaria in proportion to disease severity and were also increased in patients with *P. vivax* and *P. malariae* infections, although to a lesser extent (Nantakomol et al., 2011). Interestingly, antimalarial treatment decreased the levels of circulating EVs after 2 weeks in patients infected with *P. vivax* and *P. malariae*, but not with *P. falciparum*, suggesting that high EV levels are a marker of disease severity (Nantakomol et al., 2011).

#### 2.2.3 EVs and malaria therapy

Understanding EV fusion machinery is essential for the construction of biocompatible and tissue‐specific delivery systems aiming to leverage EVs as possible therapeutic approaches. Currently, WHO advocates the use of artemisinin‐based combination therapies to treat malaria. However, the ability of malaria parasites to acquire drug resistance highlights the need to identify novel therapeutic strategies. In this context, several studies have suggested a direct applicability of EVs to treat malaria disease. For instance, EVs derived from *P. falciparum‐*infected erythrocytes are more efficiently internalized by infected and non‐infected RBCs when compared to EVs derived from naïve erythrocytes, suggesting their potential use as a drug delivery system (Borgheti‐Cardoso et al., 2020). The same authors also demonstrated that iRBC‐derived EVs loaded with the antimalarials atovaquone and tafenoquine inhibited the erythrocytic development of *P. falciparum* better than using only iRBC‐derived EVs. Niewold et al. (2018) have described the use of immune‐modified particles combined with artesunate to target specific modulation of Ly6C^lo^ monocyte response (a monocyte subset accumulated in the brain of infected mice), which prevented mortality in a model of severe malaria. The transfer of specific microRNAs known to accumulate in sickle‐cell erythrocytes that exhibit resistance to *P. falciparum* infection, such as miR451 and let‐7i, has also been explored. Both miR451 and let‐7i, as well as miR223, have been shown to inhibit parasite growth, conferring a host defence strategy against malaria (LaMonte et al., 2012). Furthermore, average levels of plasma miR451 and miR16 are significantly lower in patients infected with *P. vivax* (Chamnanchanunt et al., 2015).

Immunization strategies using iRBC‐derived EVs collected from the peripheral blood of BALB/c mice infected with *P. yoelii* have also been explored (Martin‐Jaular et al., 2016). Successful immunization against lethal infection was accompanied by the production of IgG antibodies that recognized *P. yoelii*‐infected RBCs (Martin‐Jaular et al., 2016). Additional studies using intranasal immunization of mice with synthetic microparticles loaded with *P. vivax* peptide antigens and CpG oligodeoxynucleotide‐induced mucosal, systemic humoral and cell‐mediated immune responses (Bhat et al., 2009, 2010). Another promising strategy concerns the use of microparticles conjugated with strong adjuvant molecules to increase vaccine immunogenicity against *P. vivax* infection (Moon et al., 2012).

### 2.3 Toxoplasmosis

The apicomplexan parasite *Toxoplasma gondii* is the causative agent of a widely distributed zoonotic infection called toxoplasmosis. *T. gondii* infects humans and different animals found in all ecosystems, contaminating water, soil and food (de Barros et al., 2022). Because infection spreads readily to a variety of hosts, *T. gondii* is one of the most successful of all parasites, which makes it a serious public health problem (Suijkerbuijk et al., 2018).

In immunocompetent humans, the disease is usually asymptomatic, or may cause mild flu‐like symptoms or other nonspecific clinical signs (Dubey et al., 2012). Primary infection during pregnancy, however, can cause foetal harm or miscarriage, since parasites cross the placental barrier and infect the foetus (Dubey, 2008). In immunocompromised individuals, the disease may become severe, leading to neurological signs and, ultimately, death (Dubey & Jones, 2008; Montoya & Liesenfeld, 2004).

As an obligate intracellular parasite, *T. gondii* can infect and multiply in virtually any cell type, spreading through the lymphatic and blood systems. Tachyzoites are the active rapidly multiplying form of the parasite in the intermediate host. The chronic phase occurs about 2–3 weeks post‐infection, when extracellular tachyzoites are eliminated from host tissues and intracellular parasites differentiate into bradyzoites, a latent form surrounded by a parasitophorous vacuole and enclosed in a cyst wall, which are hard to eradicate by host immune system and drugs (de Barros et al., 2022). In the definitive hosts, ingested bradyzoites undergo multiple rounds of asexual reproduction increasing parasitaemia. Subsequently, sexual reproduction occurs leading to the production of unsporulated oocysts which are released into the environment where mature oocysts are formed by sporulation (Blader et al., 2015; Delgado Betancourt et al., 2019). The release of *Toxoplasma* material within EVs has been recognized as an important mechanism to control host‐parasite interactions.

#### 2.3.1 EVs and toxoplasmosis pathogenesis

During infection, *T. gondii* modulates the host immune response to develop long‐term protection against reinfection. The establishment of this finely regulated host‐parasite interaction governs infection and disease development. In this context, high levels of IFN‐γ control the acute and chronic phases of infection by killing the parasite or keeping it in the latent form, respectively (Dupont et al., 2012; Yap et al., 2000). Accordingly, the release of parasite antigens is essential to modulating the host immune system, although these mechanisms—as well as the role of cargo—remain to be determined.

EVs from L6 cells infected with *T. gondii* change both host and neighbouring cells’ proliferation mechanisms and alter the cell cycle, possibly by the action of miRNAs, as identified by web‐based tools (Kim et al., 2016). Studies seeking to characterize *T. gondii*‐produced EVs revealed that these particles carry virulent factors, such as proteins used by tachyzoites for invasion and replication in the host cell (Li et al., 2018a, 2018b; Quiarim et al., 2021). Indeed, these tachyzoite‐secreted EVs can induce macrophage activation, resulting in the production of proinflammatory cytokines and a reduction in IL‐10 levels (Li et al., 2018a). *T. gondii*‐infected macrophages also produce EVs carrying pathogen‐associated molecular patterns (PAMPs), which drive activation and a proinflammatory phenotype in other macrophages in the vicinity, in a TLR/MyD88‐dependent process (Bhatnagar et al., 2007).

Besides proteins, *T. gondii*‐derived EVs carry miRNAs that promote gene regulation in the host cells (Silva et al., 2018). Cong et al. demonstrated that *T. gondii* infection controls the expression of specific host microRNAs, a mechanism that correlates with an unbalanced defensive response of infected cells that leads to increasing parasite replication (Cong et al., 2017; Hakimi & Menard, 2010). During infection, elevated miR‐17‐92 miRNA and miR‐106b‐25 induce a pro‐survival pathway by subverting apoptosis in host cells containing *T. gondii* (Cai & Shen, 2017; Cai et al., 2014). Other immunomodulatory microRNAs are upregulated during infection, including miR‐146a, which regulates inflammatory response and parasite load, and miR‐155, which is involved in the recruitment of T regulatory (Treg) and CD8^+^ T cells during *T. gondii* infection (Cai & Shen, 2017; Taganov et al., 2006). The main findings concerning the role of EVs derived from *T. gondii*‐infected cells in disease pathogenesis are summarized in Figure 1.

#### 2.3.2 EVs and toxoplasmosis diagnosis

As in other parasite infections, virulent strains of *T. gondii* produce greater amounts of EVs than avirulent strains (Quiarim et al., 2021). Accordingly, da Cruz et al. (2020) detected higher concentrations of serum derived EVs in infected patients than in uninfected individuals. Likewise, in animal studies, EV concentrations were higher in infected mice than in uninfected ones (Maia et al., 2021).

Studies conducted in mice have also revealed upregulation of miR‐712‐3p, miR‐511‐5p and miR‐217‐5p in cells infected with *T. gondii* RH and ME49 strains (Cai et al., 2014). Interestingly, upregulation of these three microRNAs has not been identified in mice infected with other parasites, such as *P. berghei, P. yoelii, P. chabaudi, C. parvum*, mouse hepatitis virus or *Staphylococcus aureus*, reinforcing the specificity of this mechanism to toxoplasmosis (Jia et al., 2014). This result encourages the use of such molecules as potential early biomarkers for *T. gondii* infection.

#### 2.3.3 EVs and toxoplasmosis therapy

The release of parasite material through infected cell‐derived EVs has been recognized as an important mechanism to control host‐parasite interactions. Aline et al. (2004) were the first to describe EV production during *T. gondii* infection, demonstrating that dendritic cell‐derived EVs play a crucial role in inducing protective immunity against infection. Beauvillain et al. (2007) also indicated that EVs produced by SRDC (a CD8α^+^CD4^−^ dendritic cell line) treated with *T. gondii* antigens induced protection against infection in vivo. In syngeneic CBA/J mice, treatment reduced the number of cysts in brain tissue, while in allogeneic C57BL/6 mice, it resulted in increased survival due to strong humoral and cellular responses. Based on these observations, EVs from *T. gondii‐*pulsed dendritic cells were used as a vaccine in CBA/J mice before pregnancy, which protected pups against infection during gestation by eliciting a specific, protective T‐cell response (Beauvillain et al., 2009). EVs isolated from *T. gondii*‐infected human hepatoblastoma cells have increased protective efficacy when adsorbed to alum adjuvant (EV‐alum) in comparison to excretory secretory antigens thus adsorbed (ESA‐alum). Mouse immunization with EV‐alum before a challenge with orally administered *T. gondii* cysts significantly reduced the brain cyst burden by 75% and induced higher humoral and cellular immune responses. This finding suggests that alum‐adjuvanted EVs derived from *T. gondii*‐infected cells may provide a new perspective for toxoplasmosis vaccination (Tawfeek et al., 2023).

### 2.4 Leishmaniasis

Leishmaniasis is a group of chronic infectious diseases caused by protozoa of the genus *Leishmania* (Afrin et al., 2019). In 2022, there were approximately 30,000 cases of visceral leishmaniasis (VL) and over 200,000 cases of cutaneous leishmaniasis (CL), with the actual number estimated to exceed 1 million (WHO, 2023, www.who.int/news‐room/fact‐sheets/detail/leishmaniasis). Leishmaniasis is also the second deadliest parasitic disease worldwide, after malaria (Pace, 2014).

VL, also known as *kala‐azar*, is the most severe form of this disease, accounting for 25% of global cases (Hartley et al., 2013). In the Old World, *L. donovani* and *L. infantum* were the primary causes, while *L. infantum* is the sole etiological agent in the New World (Murray et al., 2005). The parasite migrates to the bone marrow and visceral organs, such as the liver and spleen, often leading to fatal outcomes if untreated (Hartley et al., 2013).

CL presents in three main forms: localized, diffuse and mucosal. Localized CL, the most prevalent form of CL, begins with a nodule at the site of infection that eventually ulcerates (Kaushal et al., 2023). Diffuse CL is characterized by chronic, metastatic lesions with a high parasite load, blunted immune response and poor response to treatment. The *L. mexicana* complex is responsible for diffuse CL in the Americas (Gurel et al., 2020). Mucosal leishmaniasis (ML), also called ‘metastatic’ cutaneous leishmaniasis (Kevric et al., 2015), affects 5%–10% of CL patients in the New World. It involves parasite migration to the oronasal region, often due to inadequate treatment (von Stebut & Tenzer, 2018; von Stebut et al., 2000). ML exhibits a low parasite load with heightened immune response (Hepburn, 2003), resulting in mucous membrane destruction (Jirmanus et al., 2012).

*Leishmania* RNA virus 1 (LRV1), found in certain *Viannia* subgenus species like *L. (V.) guyanensis*, is linked to severe ML cases (Atayde et al., 2019; Olivier & Zamboni, 2020). Endosomal TLR3 receptors recognize the LRV1 genome and trigger a hyperinflammatory response that results in a direct NLRP3‐related exacerbated pathological condition (de Carvalho et al., 2019).

#### 2.4.1 EVs and leishmaniasis pathogenesis

*Leishmania* parasites manipulate host immune responses to evade detection. They induce macrophage polarization, releasing pro‐ or anti‐inflammatory cytokines to facilitate their survival (Kumar et al., 2009). This manipulation varies across *Leishmania* species. For instance, *L. amazonensis* promastigotes weaken immune cell activation compared to *L. major* and *L. braziliensis* (Vargas‐Inchaustegui et al., 2008; Xin et al., 2008, 2007). *Leishmania* also modulates the adaptive immune response, inducing IL‐10 production to regulate IFN‐γ of CD4^+^ T cells in localized CL (Antonelli et al., 2005; Bottrel et al., 2001; Carneiro et al., 2009). Conversely, diffuse LC is characterized by a high production of anti‐inflammatory cytokines, such as IL‐10 and TGF‐β, with no IFN‐γ in lymphocytes from patients (Bomfim et al., 1996).

EVs play a crucial role in *Leishmania* evasion from the host immune system by bearing such cargo as GP63, elongation factor‐1α and heat shock proteins (HSP), though their composition varies among *Leishmania* species and strains (Ibarra‐Meneses et al., 2023; Tano et al., 2022). These proteins can reach the host cell cytosol and inhibit IFN‐γ signalling while impairing the expression of microbicidal factors like NO and TNF‐α (Silverman & Reiner, 2011). *L. mexicana*‐derived EVs, for instance, contain GP63, PP2C and miRNAs, which reduce NO production in macrophages, aiding parasite survival (Soto‐Serna et al., 2020). *L. infantum*‐derived EVs decrease antigen‐presenting cell activation by reducing co‐stimulatory molecule expression (Perez‐Cabezas et al., 2019). HSP100, contained in *L. donovani*‐derived EVs, inhibits the dendritic cell response to Th1 cytokines and promotes neutrophil recruitment while altering cytokine production (Silverman & Reiner, 2011; Silverman et al., 2010a, 2010b, 2013).

In *L. braziliensis*, EV‐primed macrophages showed increased production of IL‐1β, IL‐6, IL‐10 and TNF‐α upon infection, suggesting a role of EVs in exacerbating inflammatory responses. Conversely, inhibition of EV secretion reduced cytokine production and infection rates, indicating EV involvement in pathogenic inflammation (Peixoto et al., 2023). *L. major*‐derived EVs contain LmPRL, which promotes parasite growth within macrophages. These parasites have a high capacity to dampen TLR4‐related immune responses, and macrophage exposure to these exosomes promotes Th2‐related polarization (Leitherer et al., 2017; Silverman et al., 2010a, 2010b). Despite this polarization, *L. major*‐derived exosomes induced the production of IL‐17a and IL‐10 (Atayde et al., 2015, 2016). The contribution of EVs to the pathogenesis of leishmaniasis is depicted in Figure 1.

GP63 appears to be omnipresent in *Leishmania*‐derived EVs, reflecting its importance as a surface protease and a key virulence factor. da Silva Lira Filho et al. (2021) highlights the role of GP63 in enhancing inflammation during skin infection, with varying GP63 loads in *L. amazonensis*‐derived EVs affecting macrophage responses differently. Additionally, the release of EVs in the sandfly midgut and their co‐inoculation during host infection exacerbate cutaneous lesion pathology by increasing the production of cytokines, including IL‐2, IL‐4, IL‐10, IL‐17, IL‐23 and IFN‐γ (Atayde et al., 2016).

Douanne et al. (2022) demonstrated horizontal gene transfer in parasites, showing that *Leishmania* can exchange genes through EVs—including genes responsible for drug resistance. This transmission modulates promastigote fitness, reducing oxidative stress accumulation and modifying the recipient parasites’ proteome, thus promoting the spread of drug‐resistant populations. It has been proposed that LRV1 is transmitted among *Leishmania* during cell division. Atayde et al. (2019) showed that *Leishmania*‐derived EVs serve as a delivery system for LRV1, enhancing virus transmission and host infectivity. This transmission is not restricted to *Leishmania* of the same species; it can also occur interspecies (Atayde et al., 2019; Olivier & Zamboni, 2020). Additionally, Cronemberger‐Andrade et al. (2014) demonstrated that EVs from *L. amazonensis*‐infected macrophages stimulate other macrophages to produce proinflammatory cytokines, promoting Th1‐related immune responses and favouring *Leishmania* elimination. These EVs may thus carry cytokines that intensify the immune response, MHC‐antigenic peptide complexes, costimulatory molecules and unbound antigens.

#### 2.4.2 EVs and leishmaniasis diagnosis

*Leishmania*‐derived EVs hold potential for serological diagnosis of leishmaniasis. Research groups are exploring miRNAs and other biomarkers to differentiate *Leishmania* infection from other parasitic diseases, considering their increased levels in the host bloodstream (Esteves et al., 2023). However, identifying the infecting *Leishmania* spp. remains challenging.

#### 2.4.3 EVs and leishmaniasis therapy

Due to their potent immunogenicity, EVs have emerged as promising candidates for vaccines and immunotherapies against leishmaniasis. Intranasal vaccines using a total lysate of *L. amazonensis* (LaAg), LPG and partially purified extracellular serine proteases from LaAg have demonstrated partial protection against *Leishmania* spp. infections (de Matos Guedes et al., 2014; Leal et al., 2015; Oliveira‐Maciel et al., 2021). Although vaccines against leishmaniasis do exist, none is highly effective or developed for human use. Challenges in vaccine development include coverage against multiple parasite species and varied clinical manifestations, parasite complexity and low financial support.

Ramos et al. (2020) showed that EVs from adipose tissue‐derived mesenchymal stromal cells (AD‐MSCs) provided partial protection against *L. amazonensis* lesions; when this cell therapy was combined with conventional pentavalent antimonial treatment, it resulted in faster lesion healing. As most therapeutic factors present in AD‐MSCs are presumed to reside in their EVs, these vesicles may offer benefit without the downsides of cell transplantation, as well as advantages including lower immunogenicity, reduced risk of treatment rejection, and easier handling and administration.

### 2.5 Helminths

Helminths, a group of parasitic worms, pose a significant and yet neglected global health challenge, affecting both humans and animals (Jourdan et al., 2018). These parasites are categorized into Nematoda (roundworms) and Platyhelminthes (flatworms). About one‐fifth of the world's population is infected with soil‐transmitted helminths, with the number rising to one‐third when considering all helminth types. Many common human infections involve soil‐transmitted helminths, with schistosomes causing schistosomiasis and filarial worms leading to onchocerciasis and lymphatic filariasis (Hotez et al., 2008).

#### 2.5.1 EVs and helminth pathogenesis

The persistence of helminth infections is significantly influenced by their evolved strategies to modulate host immune responses, influencing a variety of cell types in the host (Maizels & McSorley, 2016). Although this interaction is well established, the role of EVs has been recognized only in the last decade as a crucial mechanism in this dynamic process (White et al., 2023). EVs serve as a conduit for parasitic helminths to transfer small RNAs and proteins directly into host cells, thereby altering intracellular pathways (Coakley et al., 2016, 2015, 2017). Interestingly, EV secretion by parasites can be blocked by the antiparasitic drug ivermectin (Loghry et al., 2020). This discovery unveils a potentially novel mechanism of action centred on the modulation of parasite‐host immune interactions and offers new insights into therapeutic strategies (Harischandra et al., 2018).

The first descriptions of EVs in helminths came from studies in *Echinostoma caproni* and *Fasciola hepatica* (Marcilla et al., 2012). Additional clarity on the immune‐regulating effects of helminth‐sourced EVs was provided through studies showing that EVs from the nematode *Heligmosomoides polygyrus* can dampen Type 2 innate immune responses and reduce the eosinophilia typically triggered by the fungal allergen *Alternaria* (Buck et al., 2014). Further studies demonstrated that the influence of helminth derived EVs on the host immune system is complex and multifaceted. Depending on the helminth species and the targeted host cells, these EVs can suppress T‐cell proliferation, induce the release of inflammatory cytokines, or promote anti‐inflammatory responses (Table 3). For instance, macrophages are highly active in EV uptake, being differentially regulated depending on the origin of helminth derived EVs. The human filarial parasite *Brugia malayi* releases EVs that trigger M1‐type macrophage activation, marked by increased production of certain immune molecules (Zamanian et al., 2015), while other nematode‐derived EVs (i.e., *Heligmosomoides polygyrus*) have immunosuppressive effects (Coakley et al., 2017). Similarly, *Fasciola hepatica*‐derived EVs were found to reduce the migratory capacity of monocytes and induce a mixed M1/M2 response, while *Dicrocoelium dendriticum*‐derived EVs did not affect monocyte migration and present proinflammatory properties (Sanchez‐Lopez et al., 2023).

**TABLE 3:** Summary of studies reporting the immunomodulatory actions of helminth‐derived EVs.

Helminth species	Key findings	Reference
*Brugia malayi*	EVs elicited a classically activated phenotype in host macrophages.	Zamanian et al. (2015)
	Ivermectin rapidly inhibited EV release by the parasite.	Harischandra et al. (2018)
	Microfilarial stage of *Brugia malayi* released EVs that were taken up by human DCs and monocytes, modulating the mTOR pathway.	
*Clonorchis sinensis* (liver fluke)	*C. sinensis*‐derived EVs promoted M1 macrophage activation and contributed to biliary injuries.	Yan et al. (2021)
	EVs induced IL‐6 and TNF‐α production in biliary epithelial cells via the TLR9‐mediated ERK pathway.	Wang et al. (2023b)
*Echinococcus granulosus*	EVs from *E. granulosus* inhibited the proliferation of CD4^+^ and CD8^+^ T‐cells, the production of inflammatory cytokines, and IL‐10.	Sanchez‐Lopez et al. (2021)
	*E. granulosus*‐derived EVs are internalized by dendritic cells (DC), inducing their maturation with increased CD86 and down‐regulation in major histocompatibility complex class II (MHC II).	Zhou et al. (2019).
	EV‐cargo derived from drug‐treated *E. granulosus* enhanced DC activation toward a type 1 proinflammatory profile against the parasite.	Nicolao et al. (2023)
	*E. granulosus* cystic fluid‐derived EVs ameliorated Th2 allergic airway inflammation through the induction of Treg cells.	Jeong et al. (2021)
	EVs from the plasma of 7‐week infected mice increased the relative abundance of Treg cells and IL‐10.	Shi et al. (2022)
	EVs induced DCs to produce proinflammatory cytokines, MHC II, as well as costimulatory molecules CD40, CD80 and CD86.	Zhang et al. (2022)
*Echinococcus multilocularis*	EVs suppressed NO production in RAW macrophages via downregulation of inducible nitric oxide synthase expression, suppressed pro‐inflammatory cytokines (IL‐1α and IL‐1β), and induced the LPS/TLR4 pathway.	Zheng et al. (2017)
	EVs released by the protoscolex promoted endothelial cell proliferation and tube formation.	Liu et al. (2023)
*Fasciola hepatica*	miRNAs with immunoregulatory, growth‐ and cancer‐related functions were highly expressed in EVs.	
	Molecular characterization of parasite‐derived EVs: molecules packaged within the EVs were highly regulated, most likely to facilitate parasite migration through host tissue and to counteract the host immune attack.	Cwiklinski et al. (2015)
	EVs from *F. hepatica* were administered to mice with dextran sulphate sodium (DSS)‐induced colitis, leading to a reduced amount of pro‐inflammatory cytokines and interfering with both MAPK and NF‐kB pathways.	Roig et al. (2018)
	380 surface proteins were identified, encompassing a variety of virulence factors, membrane transport proteins and molecules linked to the creation and transport of parasite EVs.	de la Torre‐Escudero et al. (2019)
*Heligmosomoides polygyrus*	EV internalization caused downregulation of type 1 and type 2 immune‐response‐associated molecules (IL‐6 and TNF; Ym1 and RELMα) and inhibited expression of the IL‐33 receptor subunit ST2.	Coakley et al. (2017)
	EVs secreted by *H. polygyrus* displayed a unique lipid content that was involved in immunomodulatory actions.	Simbari et al. (2016)
	EVs from *H. polygyrus* dampened Type 2 innate responses and reduced eosinophilia typically triggered by the fungal allergen *Alternaria*.	Buck et al. (2014)
*Nippostrongylus brasiliensis* (rodent model for human hookworm infection)	*N. brasiliensis*‐derived EVs were used as treatment in a mouse model of chemical‐induced colitis and demonstrated to reduce IL‐6, IL‐1β, IFNγ and IL‐17a and increase IL‐10 expression.	Eichenberger et al. (2018)
*Schistosoma japonicum*	*S. japonicum*‐derived EVs were internalized by mammalian cells and transferred their associated miRNAs to recipient cells.	Zhu et al. (2016)
	*S. japonicum*‐derived EVs stimulated the murine macrophage‐like cell line RAW264.7 to produce NO alongside other indicators of a Type 1 pathway.	Kalra et al. (2016)
	*S. japonicum*‐derived EVs promoted M1‐type polarization and increased pro‐inflammatory cytokines such as TNF‐α and IL‐12 in RAW264.7	Wang et al. (2015)
	*S. japonicum*‐derived EVs were primarily taken up by macrophages and other immune cells in the host's peripheral blood. The microRNA contents of these EVs, specifically miR‐125b, were transferred to the recipient cells, resulting in enhanced macrophage proliferation and increased production of TNF‐α.	Liu et al. (2020)
	Egg‐derived EVs of *S. japonicum* carried a miRNA‐33 that promoted liver fibrosis.	Wang et al. (2022b)
*Schistosoma mansoni*	Schistosome miRNAs were characterized and found in circulating exosomes from infected mice.	
	*S. mansoni*‐derived EVs interfered with Th2 cell differentiation by transferring miR‐10, which targets MAP3K7, thereby reducing the activity of NF‐κB, a key factor in Th2 cell differentiation and function.	Meningher et al. (2020)
	Proteomic analysis of *S. mansoni*‐derived EVs revealed a collection of proteins with known or potential relevance in host‐parasite communication, such as proteases and antioxidants.	Kifle et al. (2020b)
	*S. mansoni*‐derived EVs were taken up by vascular endothelial cells and monocytes, leading to a gene expression signature associated with intravascular parasitism, as well as immune cell trafficking and signalling.	Kifle et al. (2020a)
	*S. mansoni*‐derived EVs increased expression of IL‐12 and IL‐10 by dendritic cells through interaction with DC‐SIGN.	Kuipers et al. (2020)
*Taenia pisiformis*	*T. pisiformis* cysticercus‐derived vesicles induced the production of IL‐4, IL‐6, IL‐10, IL‐13 and Arg‐1 and downregulated the expression of IL‐12, IFN‐γ and iNOS in RAW264.7 cells.	Wang et al. (2020)
*Trichinella spiralis*	*T. spiralis*‐derived EVs exhibited immunomodulatory properties in peripheral blood mononuclear cells (increased IL‐10 and IL‐6; decreased IL‐17a).	Kosanovic et al. (2019)
	*T. spiralis*‐derived EVs increased Th2 and Treg differentiation and ameliorated 2,4,6‐trinitrobenzenesulfonic acid (TNBS)‐induced colitis in mice.	Yang et al. (2020)
	*T. spiralis*‐derived EVs were administered to mice with dextran sulphate sodium (DSS)‐induced colitis and increased the infiltration of M2 macrophages in the colon.	Gao et al. (2021)
	EVs from *T. spiralis* larvae carried miR‐1‐3p and let‐7‐5p, which induced the polarization of bone‐marrow macrophages toward the M2b type and suppressed the activation of fibroblasts.	Wu et al. (2022)
*Trichuris muris* (rodent model for human roundworm infection)	EVs isolated from *Trichuris muris* excretory/secretory products (ES) induced protective immunity in mice.	Shears et al. (2018)

Parasite‐related soluble antigens may also alter the profile of macrophage‐derived EVs, highlighting another mechanism of EV‐related immunomodulation (Zakeri et al., 2021). In addition, helminth derived EVs have been shown to reprogram host gut, liver and lung cells to create a more favourable environment for the parasite (Ruangsuwast et al., 2023; Wang et al., 2023a, 2022a). This highlights the complexity of the interaction between nematode‐derived EVs and host cells, varying significantly based on the specific nematode species and the host cells involved. This intricate, finely tuned interplay underscores the sophisticated nature of helminth‐host interactions and the vital role of EVs in these dynamics. A notable finding is that antiparasitic treatments can alter the characteristics of parasite EVs, as recently highlighted in a study where EVs collected from patients receiving antiparasitic therapy exhibited significant changes compared to those from untreated controls. Notably, these altered EVs elicited a stronger proinflammatory response against the parasite (Nicolao et al., 2023). The contribution of EVs to the pathogenesis of helminths is shown in Figure 1.

#### 2.5.2 EVs in helminthiasis diagnosis and therapy

The potential applications of helminth‐derived EVs extend their significance beyond just host‐parasite dynamics, showing promise in broader medical applications. They appear to have potential in diagnostic and therapeutic fields (Ashour et al., 2022; Gao et al., 2022; Guo et al., 2022; Sotillo et al., 2016). The unique molecular signatures of helminth specific EVs and their stability in body fluids suggest a potential role as novel biomarkers, potentially transforming infection detection and monitoring and allowing earlier and more accurate diagnoses (Meningher et al., 2017; Mu et al., 2021). Additionally, the immunomodulatory properties of these EVs are attracting attention for their prospective roles in developing vaccines and treating autoimmune disorders, opening new possibilities for leveraging these vesicles in therapeutic strategies (Zwiernik et al., 2019).

## 3 CONCLUSIONS AND PERSPECTIVES

This review highlights a perspective into the complex world of parasite‐released EVs, providing insights into their physical and biochemical functions. Such information is particularly relevant to maintaining accurate methods, detecting biomarkers and developing medicines, and is essential in ensuring robust repeatability across laboratories and reducing potential artifact production and misinterpretation errors.

EVs have critical functions in numerous species, permitting parasite cells to communicate with other cells via strategies such as horizontal gene transfer and interspecies communication. Interestingly, biomarkers from parasite‐derived EVs differ from those detected in host cells, highlighting the need to consider parasite developmental stage and host type (invertebrate or vertebrate). Finally, the discovery of novel EV‐enriched biomarkers can provide valuable information for the development of future immunizations as well as for the diagnosis and prevention of parasitic infections, thus representing an enormous step forward in the field.

## AUTHOR CONTRIBUTIONS

Ana Acacia Sá Pinheiro and Ana Claudia Torrecillas: conceptualization; methodology; visualization; writing—original draft; writing—review and editing. Bruno Solano de Freitas Souza, Fernanda Ferreira Cruz, Herbert Leonel de Matos Guedes, and Tadeu Diniz Ramos: conceptualization; methodology; visualization; writing—review and editing. Miqueias Lopes‐Pacheco and Celso Caruso‐Neves: methodology; visualization; writing—review and editing. Patricia R. M. Rocco: conceptualization; funding acquisition; methodology; supervision; visualization; writing—original draft; writing—review and editing.

## CONFLICT OF INTEREST STATEMENT

The authors declared no conflict of interest.
